# May-Thurner Syndrome: Is It on Our Radar?

**DOI:** 10.7759/cureus.82638

**Published:** 2025-04-20

**Authors:** Moazzma Ifzaal, Saba B Bawan, Danielle L Dsouza, Olufunbi Kupoluyi, Ahmed Malik, Muhammad Uzair Ilyas, Hifza Habib, Abdul Manan

**Affiliations:** 1 Acute Medicine, University Hospitals of North Midlands (UHNM), Royal Stoke University Hospital, Stoke-on-Trent, GBR; 2 Emergency Department, University Hospitals of North Midlands (UHNM), Royal Stoke University Hospital, Stoke-on-Trent, GBR; 3 Internal Medicine, Arrowe Park Hospital, Birkenhead, GBR; 4 Respiratory Medicine, University Hospitals of North Midlands (UHNM), Royal Stoke University Hospital, Stoke-on-Trent, GBR

**Keywords:** computed tomography venography (ctv), deep vein thrombosis (dvt), inferior vena cava (ivc), may-thurner syndrome (mts), pulmonary embolism (pe)

## Abstract

May-Thurner syndrome (MTS) is a vascular condition in which the right iliac artery compresses the left iliac vein against the lumbar spine. MTS can present as either acute or chronic deep vein thrombosis (DVT), which may lead to pulmonary embolism (PE). We present the case of a 61-year-old female with a medical history of unprovoked DVT on rivaroxaban, who presented with pain and swelling of the left lower limb, associated with shortness of breath for two weeks. Radiological investigations confirmed DVT with unilateral MTS involving the left common iliac vein.

Therefore, MTS should be considered as a differential diagnosis in older women presenting with DVT and PE, particularly in cases of recurrence or other unprovoked thromboembolic events.

## Introduction

May-Thurner syndrome (MTS) was first observed by Rudolf Virchow in the early 1850s, when he noted an increased incidence of left iliofemoral vein compression by the right common iliac artery in cadaveric studies of patients with left iliofemoral thrombosis. The condition was later formally described by May and Thurner in 1957 through studies on multiple cadavers [1]. MTS is a progressive condition with long-term disabling complications, so failing to address the underlying anatomic lesion may result in recurrence and potentially life-threatening complications such as iliac vein rupture, pulmonary embolism, and post-thrombotic syndrome [2]. The prevalence of MTS in the general population ranges from 14% to 32%, with a higher incidence among women, particularly between their second and fourth decades of life. However, among patients presenting with symptomatic lower-extremity venous disorders, MTS is identified as the underlying cause in only 2-5% of cases [3]. Diagnosis is typically confirmed through CT imaging and iliac venography [4]. The treatment approach includes thrombolysis, anticoagulation, stenting of the left iliac vein, and placement of an inferior vena cava (IVC) filter. Anticoagulation alone is insufficient to prevent recurrence [5].

## Case presentation

A 61-year-old female with a history of unprovoked deep vein thrombosis (DVT) on anticoagulation presented in December with a two-week history of sudden cramp-like pain in her left lower limb, accompanied by swelling, stiffness, tingling, and difficulty walking. She also reported episodes of shortness of breath, fatigue, and stabbing chest pain occurring 3-6 times per day over the same period. Additionally, she had been experiencing persistent lower back and left hip pain for 6-7 weeks. She noted difficulty swallowing food, which was associated with a non-productive cough, but reported no symptoms of recent chest infection, travel, or surgery. On examination in the ED, she was alert, afebrile, and hypotensive (blood pressure (BP) 97/55 mmHg). Her left lower limb was swollen, tender, and purple in colour from the hip to the toes. The pedal pulses were intact, but sensation in the left foot was reduced. The mid-calf of the left leg was swollen and tender over the deep venous system distribution on palpation.

Table 1 outlines detailed investigations of the patient on admission [1]. Blood tests showed a markedly elevated D-dimer, raising suspicion for a thrombotic event such as PE or DVT.

**Table 1:** Results of laboratory investigations on patient’s admission. eGFR: Estimated glomerular filtration rate; INR: International normalized ratio; APTT: Activated partial thromboplastin time.

Laboratory Investigations	Result on Admission	Normal Range
Haemoglobin	131	115-165 g/L
Sodium	141	133-146 mmol/L
Potassium	4.4	3.5-5.3 mmol/L
Urea	4.9	2.5-7.8 mmol/L
Creatinine	112	45-84 µmol/L
eGFR	46	>90 mL/min
C-reactive protein (CRP)	74	0-5 mg/L
Troponin	6	0-39 ng/L
INR	1	0.8-1.2
APTT ratio	0.79	0.8-1.17
D-dimer	22,876	<499 ng/mL

**Figure 1:**
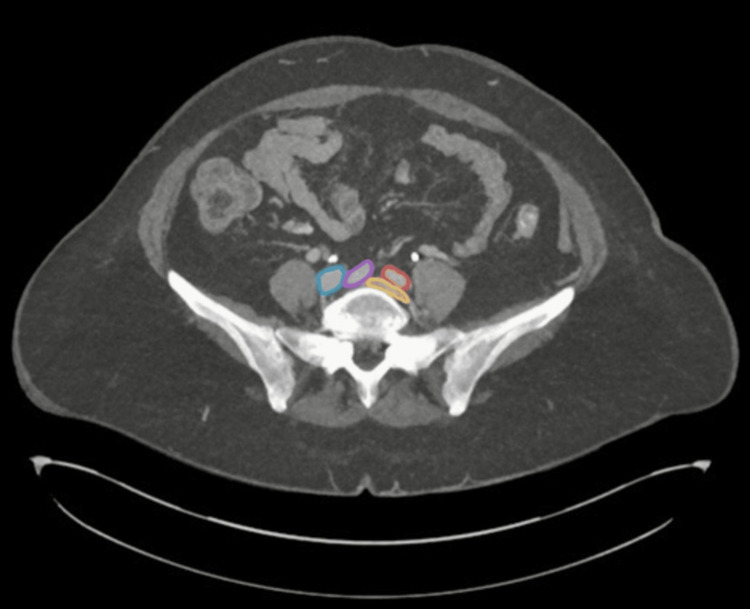
Axial view of pelvic computed tomography venography. The image demonstrates compression of the left common iliac vein (outlined in yellow) by the right common iliac artery (outlined in purple) as it crosses the lumbosacral promontory, classical for May-Thurner syndrome. (Color-coded illustration of arteries and veins: right common iliac vein – blue; right common iliac artery – purple; left common iliac artery – red; left common iliac vein – yellow.)

**Figure 2:**
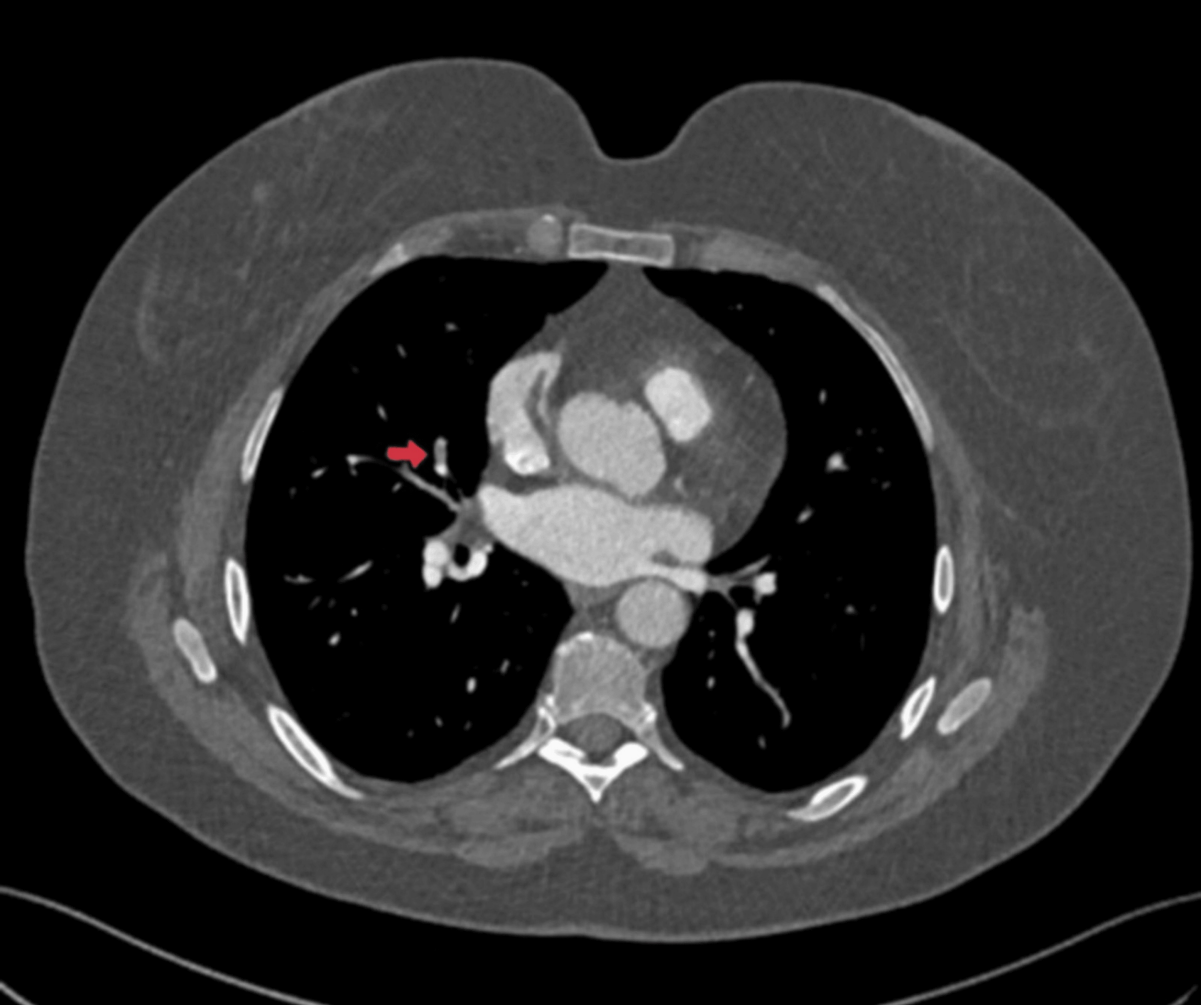
Axial CTPA image showing a pulmonary embolism in the middle lobe of the right lung (red arrow). CTPA: Computed Tomography Pulmonary Angiography.

A computed tomography venogram (CTV) of the lower limbs showed an extensive thrombus involving the left distal common iliac, full-length external iliac, internal iliac, common femoral, profunda femoris, superficial femoral, popliteal, and long saphenous veins. The left common iliac vein outflow was compressed by the right common iliac artery, indicating MTS, as shown in Figure 1. Subtle fat stranding around the thrombosed veins suggested concurrent thrombophlebitis. A subsequent computed tomography pulmonary angiography (CTPA) confirmed a segmental PE in the middle lobe, as shown in Figure 2.

## Discussion

MTS derives its name from researchers Dr. Robert May and Dr. Joseph Thurner, who sought to understand why pelvic vein thrombosis was more common on the left side. Numerous studies indicate that MTS is an infrequently diagnosed vascular disorder [6]. Data from several autopsy studies throughout the 1900s identified findings consistent with MTS in approximately 20% of cadavers in an unselected population [7]. As highlighted in this case report, the symptoms may be associated with unilateral and recurrent DVTs, typically in the left leg, due to the anatomical transverse position of the left common iliac vein (CIV), which predisposes it to compression by the overlying right common iliac artery. However, MTS can often be asymptomatic, may uncommonly present in the right leg, and can even manifest variably with vague symptoms such as lower back or hip pain, posing further clinical and diagnostic challenges [8]. There are no standard diagnostic criteria for MTS yet. Presently, the diagnosis is determined by both clinical and imaging indicators [9]. Doppler vascular ultrasonography is a simple, convenient, and low-cost method for diagnosing DVT, while contrast-enhanced CT, CTV, or magnetic resonance venography (MRV) are more sensitive non-invasive modalities. Intravascular ultrasound remains the gold standard [10]. The management of MTS largely relates to the management of venous thromboembolism (VTE) if already present, along with targeted therapies to reduce future VTE risk. Conservative management with compression stockings is recommended for asymptomatic and non-thrombotic patients, and in cases where other interventions are high-risk [11]. Post-thrombotic syndrome (PTS) can significantly affect quality of life, and evidence suggests that catheter-directed thrombolysis does not reduce the risk of PTS but increases the risk of bleeding [12]. In our case, we decided to proceed with rivaroxaban only.

## Conclusions

MTS is a diagnostically challenging yet clinically significant vascular condition that necessitates a high index of suspicion, particularly in patients, often older women, presenting with recurrent or unexplained unilateral DVT without obvious provoking factors. This case highlights the crucial role of anatomical predisposition in thromboembolic disease and underscores the limitations of relying solely on anticoagulation for management. Instead, a comprehensive, multimodal diagnostic approach, including advanced imaging modalities such as CTV and intravascular ultrasound, is essential to accurately identify the condition and guide treatment. Definitive interventions, such as venous stenting or IVC filter placement, may be required to address the underlying mechanical obstruction and prevent recurrence, while conservative measures like compression therapy may serve as adjuncts in select cases. Ultimately, this case reinforces the importance of individualized, anatomy-informed care and contributes to the growing recognition of MTS as a critical differential diagnosis in venous thromboembolism, with implications for both immediate management and long-term outcomes.
